# Repeated gestational exposure to diesel engine exhaust affects the fetal olfactory system and alters olfactory-based behavior in rabbit offspring

**DOI:** 10.1186/s12989-018-0288-7

**Published:** 2019-01-17

**Authors:** Estefanía Bernal-Meléndez, Marie-Christine Lacroix, Pascaline Bouillaud, Jacques Callebert, Benoit Olivier, Marie-Annick Persuy, Didier Durieux, Delphine Rousseau-Ralliard, Josiane Aioun, Flemming Cassee, Anne Couturier-Tarrade, Sarah Valentino, Pascale Chavatte-Palmer, Henri Schroeder, Christine Baly

**Affiliations:** 1 NeuroBiologie de l’Olfaction, INRA, Université Paris-Saclay, 78350 Jouy-en-Josas, France; 2 CALBINOTOX, Université de Lorraine, EA7488 Vandœuvre-lès-Nancy, France; 3 Service de Biochimie et Biologie Moléculaire, Hôpital Lariboisière, Paris, France; 4 UMR BDR, INRA, ENVA, Université Paris Saclay, 78350 Jouy-en-Josas, France; 5 Center for Sustainability, Environment and Health, National Institute for Public Health and the Environment, Bilthoven, Netherlands; 6 Institute of Risk Assessment Sciences, Utrecht University, Utrecht, Netherlands

**Keywords:** Airborne pollution, Diesel exhaust, Nano-particulate matter, Gestational exposure, Olfactory dysfunction, Olfactory toxicity, Bulbar neurotransmitter disturbances, Pheromonal response, Olfactory-based behavior

## Abstract

**Background:**

Airborne pollution, especially from diesel exhaust (DE), is known to have a negative effect on the central nervous system in exposed human populations. However, the consequences of gestational exposure to DE on the fetal brain remain poorly explored, with various effects depending on the conditions of exposure, as well as little information on early developmental stages. We investigated the short-term effects of indirect DE exposure throughout gestation on the developing brain using a rabbit model. We analyzed fetal olfactory tissues at the end of gestation and tested behaviors relevant to pups’ survival at birth.

Pregnant dams were exposed by nose-only inhalation to either clean air or DE with a content of particles (DEP) adjusted to 1 mg/m^3^ by diluting engine exhaust, for 2 h/day, 5 days/week, from gestational day 3 (GD3) to day 27 (GD27). At GD28, fetal olfactory mucosa, olfactory bulbs and whole brains were collected for anatomical and neurochemical measurements. At postnatal day 2 (PND2), pups born from another group of exposed or control female were examined for their odor-guided behavior in response to the presentation of the rabbit mammary pheromone 2-methyl-3-butyn-2-ol (2MB2).

**Results:**

At GD28, nano-sized particles were observed in cilia and cytoplasm of the olfactory sensory neurons in the olfactory mucosa and in the cytoplasm of periglomerular cells in the olfactory bulbs of exposed fetuses. Moreover, cellular and axonal hypertrophies were observed throughout olfactory tissues. Concomitantly, fetal serotoninergic and dopaminergic systems were affected in the olfactory bulbs. Moreover, the neuromodulatory homeostasis was disturbed in a sex-dependent manner in olfactory tissues. At birth, the olfactory sensitivity to 2MB2 was reduced in exposed PND2 pups.

**Conclusion:**

Gestational exposure to DE alters olfactory tissues and affects monoaminergic neurotransmission in fetuses’ olfactory bulbs, resulting in an alteration of olfactory-based behaviors at birth. Considering the anatomical and functional continuum between the olfactory system and other brain structures, and due to the importance of monoamine neurotransmission in the plasticity of neural circuits, such alterations could participate to disturbances in higher integrative structures, with possible long-term neurobehavioral consequences.

## Background

Air pollution is a mixture of several components, among which particulate matter (PM) is believed to be the most widespread threat to human health and has been heavily implicated in various human diseases [1–3]. Among these components, diesel exhaust particles (DEP) represent a major source of PM-polluted air in urban environments (reviewed in [4, 5]). Several lines of research have led to the concern that the central nervous system (CNS) represents a relevant target for such particles through chronic inflammation, disruption of the blood-brain barrier and microglia activation, which may contribute to CNS diseases [6–10]. Even though few studies have examined the toxic effects following a controlled exposure to DE in humans [11, 12], increasing epidemiological evidence, as well as experimental and in vitro studies, have demonstrated that direct exposure to air pollutants lead to adverse neuropsychological effects such as decreased cognitive function, depressive symptoms, and neurophysiological disturbances, including olfactory dysfunction and auditory deficits [8, 13–17], along with early hallmarks of neurodegenerative diseases such as Alzheimer’s disease (AD) and Parkinson’s disease (PD) in elders [16–20]. Emerging data indicate that children may be particularly susceptible to air pollution-induced neurotoxicity, given their relatively immature detoxification mechanisms [13, 19, 21–26]. Consequently, gestational exposure to airborne pollution may affect fetal brain development during critical phases and might predispose the individual to neurodevelopmental and neurodegenerative diseases later in life [19].

In the last decade, epidemiological studies have highlighted a possible role of gestational exposure to air pollution in the onset of neurodevelopmental disorders, including autistic spectrum and attention deficit hyperactivity disorders, along with other learning and behavioral disabilities [24–32]. Moreover, experimental studies have shown that gestational exposure to ambient PM induces oxidative stress and inflammation as well as structural and molecular alterations of neurons in brain tissues, that might lead to postnatal neurobehavioral disorders [33–38]. Indeed, DE exposure is related to apoptotic and “swelling” phenomena in offspring neural cells and astrocyte end-feet surrounding brain capillaries, supporting the hypothesis of physiopathological abnormalities and the subsequent neurobehavioral disorders [37, 39, 40].

Interestingly, imbalances in the monoaminergic systems related to various neurocognitive disorders have also been noticed. Studies performed by Yokota et al. in mice [41–43] pointed out that maternal exposure to DE leads to perturbations of the offspring’s dopaminergic, noradrenergic and serotoninergic systems in specific brain regions that could be related to motor coordination and impulsive behavior disturbances. Nevertheless, some incongruence remains as another study has revealed contradictory monoaminergic changes [44], possibly based on different mixtures or routes used for exposure. Moreover, there are still some controversies on their effects during fetal life on cognitive function. Indeed, one study examining mice exposed to DEP in utero showed deficits in the Morris water maze test, but no differences in the passive avoidance learning test [45], whereas other studies reported no effect on learning and memory tasks [36, 46]. Furthermore, studies have shown that DE, DEP, black carbon, or NO_2_ exposure during pregnancy in mice could cause motor deficits in offspring and affect central glutamatergic and dopaminergic neurochemistry in a sex-specific manner [41, 44, 45, 47, 48].

Interestingly, olfactory dysfunction, which is a very common feature in numerous neurodegenerative disorders (such as AD and PD) preceding the cognitive and motor symptoms (reviewed by [49, 50]), has been described after direct exposure to air pollution in rodents, dogs and humans [13, 15, 51–53]. The studies conducted to date have shown some hallmarks of AD-like and PD-like neuropathology in human olfactory bulbs (OB), as reflected by neuroinflammation, oxidative stress, DNA damage, and up-regulation of neurodegenerative-disease markers [15, 52]. Post-mortem studies in humans have identified the accumulation of ultrafine particular matter (UFPM, i.e., particles with mean diameters < 100 nm) and inflammatory mediators in olfactory mucosa (OM) and OBs of children and young adults from highly polluted areas of Mexico City [13, 51, 53], suggesting that olfactory pathologies may be considered as a reliable early marker, indicative of disturbances induced in higher integrative brain structures [49, 50, 53–55]. Nevertheless, the mechanisms linking olfactory dysfunction, pollution and the development of neurodegenerative disorders are incompletely understood. Direct effect of nanoparticles in the olfactory system as well as indirect effects or both could be responsible for the observed phenotypes. Indeed, neurodegenerative disorders have been associated with protein aggregate accumulation in the olfactory system [15, 52, 56, 57] and modification of the number of olfactory dopaminergic neurons in the OB [58–60], but have also been linked to alterations in the cholinergic, noradrenergic, and serotoninergic neuromodulatory systems innervating olfactory structures [49, 55, 61].

Animal research has confirmed and expanded the observed findings in humans, and has shown that the fetal stage is a critical period of vulnerability. Nevertheless, the effects on the CNS of a gestational exposure to air pollution at such a critical period for its anatomic and functional development remain questioned and have been poorly investigated in controlled conditions mimicking indirect human exposure and in early postnatal stages [39–43]. Moreover, how the neuroanatomical continuum between brain and olfactory tissues is impacted by DE exposure remains largely unknown.

To investigate these aspects, we took advantage of a rabbit model recently developped to analyse the toxicity of repeated in utero DE exposure on the foetoplacental development, and we used a controlled inhalation system delivering a polluted air enriched in NP of small size (< 500 nm) and with a low concentration of non-inert gases [62]. The rabbit was used because of its hemodichorial placentation which is anatomically and functionally closer to that in humans than that in rodents [36, 37, 62, 63]. Based on this controlled nose-only inhalation study, previous studies have demonstrated that the feto-placental development is disturbed by maternal exposure to DE, with translocation of nanoparticle-like structures, most probably originating from the diesel exhaust, from the dams to the fetal systemic circulation across placenta [62, 64]. We investigated the olfactory system as a peripheral highly vascularized tissue at the interface between the maternal environment and the brain, as well as an indicator of disturbances in higher integrative brain structures. Our hypothesis was that the maternal inhaled DE could reach the fetal olfactory pathway, either through the hematogenic pathway or through the OM (in contact with the amniotic fluid), then the OB (throughout nerve bundles), thus resulting in brain penetration of nanoparticles with potential early neurotoxic effects. To better characterize events of predisposition to neurodevelopmental disorders, investigations focused on the early stages of development.

## Results

### Transmission Electron microscopy (TEM) analysis

#### Structural and ultrastructural changes in the OM

**Fig. 1:**
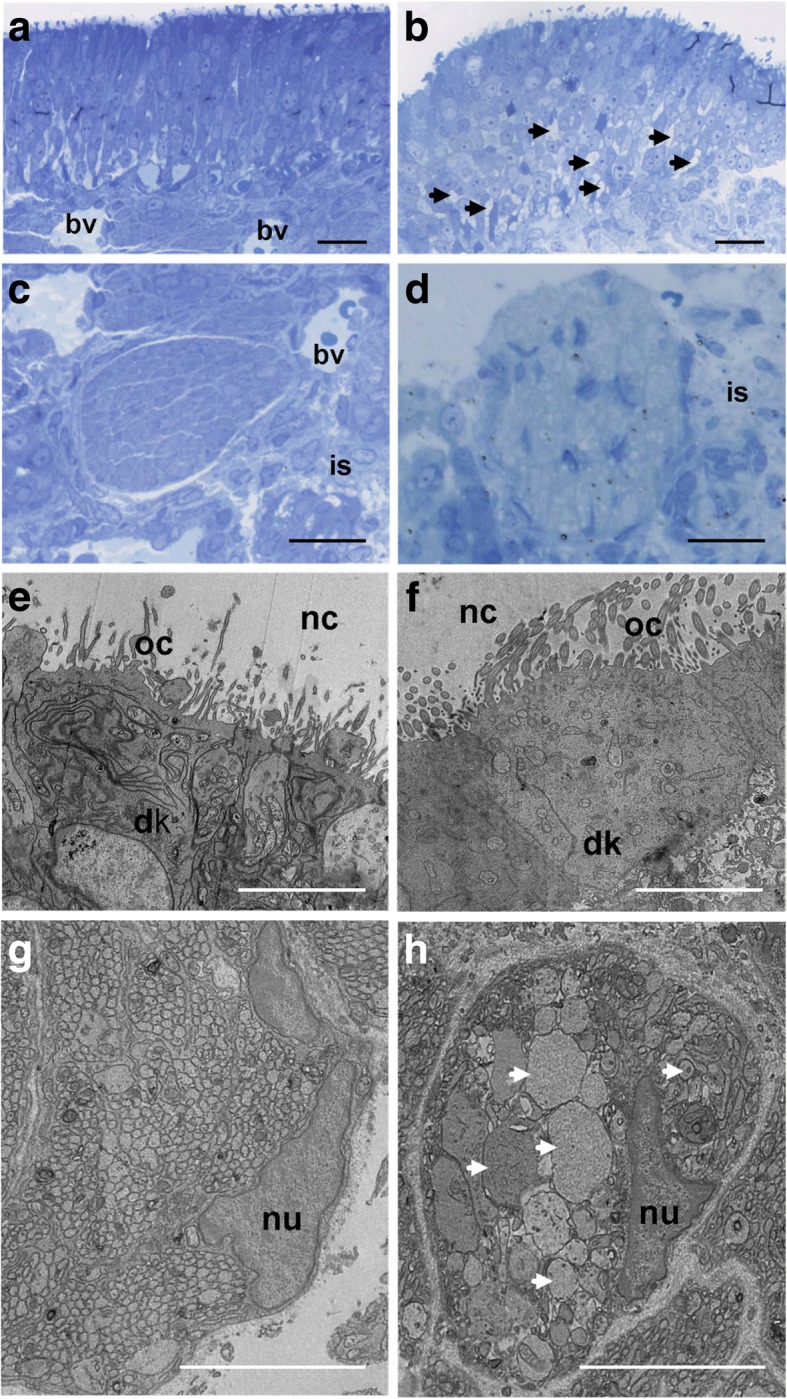
Effects of DE exposure on the olfactory mucosa. **a** to **d**. Micrograph of an olfactory mucosa from control (**a**, **c**) and exposed (**b**, **d**) GD28 fetuses. The histological coloration reveals the anatomical alterations of the olfactory mucosa (**b**) and sub-mucosa (**d**) in exposed GD28 fetuses, compared to control GD28 fetuses (**a** and **c**, respectively). Scale bar = 25 μm. **e** to **h**. Electron micrograph of dendritic knobs of an olfactory sensory neuron and of an olfactory axon bundle from control (**e** and **g**) or exposed (**f** and **h**) GD28 fetuses. Note the decrease of cell organelles in the dendrite knobs of the OSN in exposed GD28 fetuses (**f**), compared to control GD28 fetuses (**e**). The white arrows indicate axonal hypertrophy of the olfactory axons of exposed fetuses (**h**), not observed in control fetuses (**g**). Note the condensation and marginalization of the chromatin of the ensheating cells of exposed fetuses when compared to controls. Scale bar = 5 μm. bv = blood vessels; nc: nasal cavity; dk = dendritic knok; oc = olfactory cilia; nu = nucleus, is = intercellular space

Observations were made at different levels of the OM, from the apical part of the epithelium in contact with the mucus to the axon fascicles under the *lamina propria* (Fig. 1). OM of control fetuses exhibited a normal general morphology, with a pseudo-stratified columnar organization, a dense submucosal layer with blood vessels (Fig. 1a) and the presence of numerous organized nerve bundle sections (Fig. 1c). Even though exposed fetuses showed an OM of normal thickness, they exhibited relative disorganization of the epithelial cell layer, with several empty spaces visible at the basal level (Fig. 1b, see black arrows). The fasciculation of axons under the *lamina propria* was also impacted with axon bundle sections appearing usually less compact (Fig. 1d compared to Fig. 1c). The morphological alterations were noticed in all samples of exposed fetuses, but their severity was variable among zones.

At the ultrastructural level, despite comparable cilia density between exposed and control fetuses at the apical part of the OM, altered cell organelle density was observed in the dendritic endings of some exposed fetuses’ olfactory sensory neurons (OSN) (Fig. 1f compared to 1e). Under the *lamina propria*, hypertrophy of axons in the nerve bundle sections was noticed in all exposed fetuses (Fig. 1d compared to 1c). These alterations were confirmed at higher magnification (Fig. 1h, see white arrows). Chromatin was condensed and marginalized in the olfactory ensheating cells (OEC), as shown by the presence of dense patches of chromatin at the periphery of the nucleus, which was not observed in control samples (Fig. 1h compared to 1 g).

#### Structural and ultrastructural changes in the OB

**Fig. 2:**
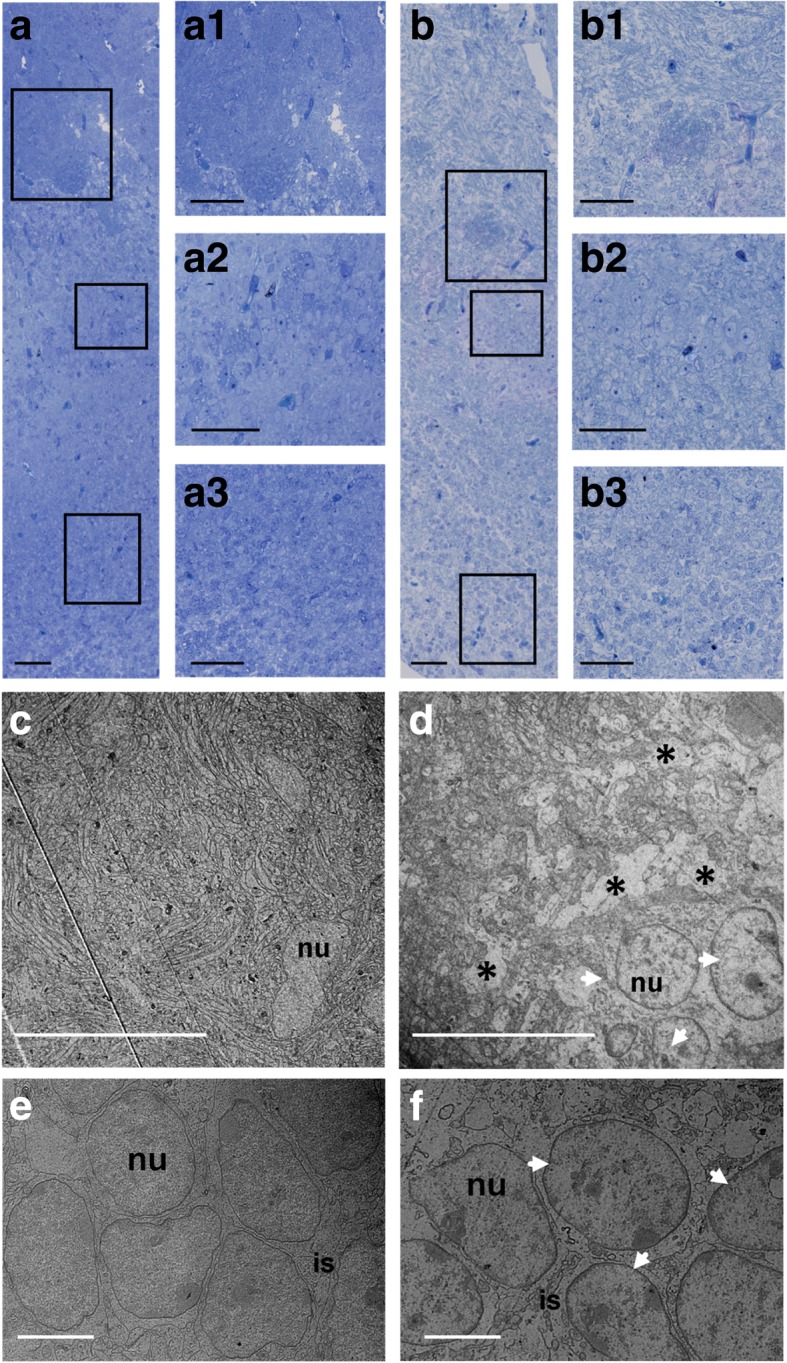
Effects of DE exposure on the olfactory bulb. **a** and **b**. Micrograph of an olfactory bulb section from control (**a**) and exposed (**b**) GD28 fetuses. The histological coloration reveals the anatomical alterations of the different layers of the olfactory bulbs of exposed GD28 fetuses (**b**), compared to control ones (**a**) and the decrease in axonal ends and the presence of areas empty of biological material around glomeruli of exposed fetuses (**b** and **b1**) when compared to controls (**a** and **a1**). Granule cells clusters seem disrupted on exposed fetuses (**b3**) when compared to controls (**a3**). No anatomical differences in the mitral (Mt) cell layer of exposed fetuses (**b2**) and control (**a2**) fetuses were observed. Scale bar: 50 μm. **c** and **d**. Electron micrograph of the glomerular layer of olfactory bulbs from control (**c**) and exposed (**d**) GD28 fetuses. Note a significant decrease in axonal endings and the presence of empty areas (black stars) around the glomerulus from exposed fetuses (**d**), compared to the glomerulus from control fetuses (**c**). The PG cells of exposed fetuses exhibit a hypertrophy and a condensation and marginalization of their chromatin (white arrows) compared to PG cells from control fetuses. Scale bar: 20 μm. **e** and **f**. Electron micrograph of the granular layer of olfactory bulbs from control and exposed GD28 fetuses. Note the disruption of granular cells clusters, along with a condensation and marginalization of their chromatin, in exposed fetuses (**f**), compared to the granular clusters from control fetuses (**e**). Scale bar: 5 μm. nu = nucleus, is = intercellular space

TEM observations were performed on each of the three stratified zones of the OB (i.e., the glomerular, the mitral and the granular cell layers) (Fig. 2). When compared to controls, where no morphological changes were observed (Fig. 2a), exposed fetuses exhibited a decrease in the density of axonal endings associated with the presence of areas empty of biological material around glomeruli (Fig. 2b1 and Fig. 2d, see black stars). The periglomerular cells (PG) exhibited cellular hypertrophy, suggesting a “swelling” phenomenon, and their nuclei displayed condensation and marginalization of the chromatin (Fig. 2d, see white arrows). Moreover, the cyto-architecture of the granule cell (Gr) layer, which normally tends to be spatially organized in restricted and well-defined clusters of potentiated mitral–granule cell synapses [65, 66], was altered. Indeed, exposed fetuses showed a disruption of the Gr clusters (Fig. 2b3 and Fig. 2f), along with condensation and marginalization of the chromatin in Gr nuclei (Fig. 2f, see white arrows). No major neuroanatomical alteration in the mitral (Mt) cell layer of exposed fetuses was observed (Fig. 2b2 compared to Fig. 2a2).

#### Presence of NP-like particles in the olfactory tissues

**Fig. 3:**
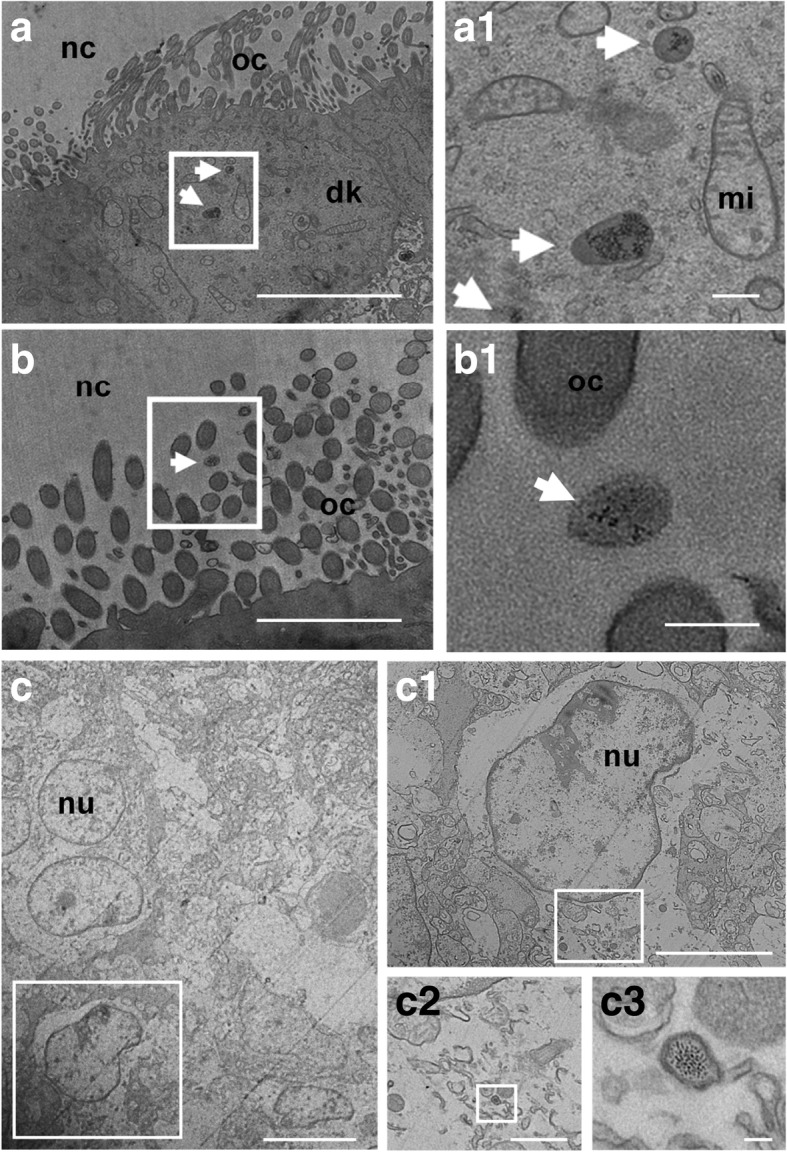
Electron micrograph showing the presence of particle-like structures in exposed GD28 fetuses’ olfactory tissues. **a** and **b**. Electron micrograph of the apical zone of the olfactory epithelium from exposed GD28 fetuses. **a.** Olfactory sensory neurons cilia with the structure in the framework enlarged at higher magnification (**a1**). The white arrows point out isolated particle-like structures (15–20 nm in diameter), which appeared inside olfactory cilia or in contact with olfactory cilia. **b.** Olfactory sensory neurons cytoplasm, with the structure in the white framework at higher magnification (**b1**). The white arrows point out lysosome-like vesicles containing NP-like particles (15–20 nm in diameter). Scale bars: a and b = 5 μm; a1 and b1 = 200 nm. **c**. Electron micrograph of the olfactory glomerulus from an exposed fetus showing the presence of NP-like particles in the cytoplasm of a PG cell. **c1**, **c2** and **c3** are enlargements at higher magnification to point out lysosome-like vesicles containing NP-like particles (5 nm in diameter). Scale bars: c. = 10 μm; c1 = 5 μm; c2 = 1 μm; c3 = 50 nm. nc = nasal cavity; dk = dendritic knok; oc = olfactory cilia; nu = nucleus, mi = mitochondrion

To determine whether inhaled diesel nanoparticles were able to reach fetuses and alter olfactory tissues before birth, ultrathin sections of OM and OB, collected at gestational day 28 (GD28), were examined by TEM. Particle-like structures (15–20 nm in diameter) were observed in OM of fetuses from which the mothers were exposed to DE. When present, they were predominantly located in cilia (Fig. 3a) or in the cytoplasm of dendritic knobs (Fig. 3b) of OSN. Interestingly, in cilia, the particle-like structures were found as single, i.e. isolated, particles (Fig. 3b1, white arrows), whereas in the cytoplasm of dendritic knobs, they were concentrated into lysosome-like vesicles (Fig. 3a and 3a1, white arrows). Particle-like structures were not visualized in OEC or OSN axons of exposed fetuses (not shown). In the OB, smaller NP-like particles were observed in lysosome-like vesicles in the cytoplasm of PG cells of exposed fetuses (5 nm in diameter) (Fig. 3c). No particle-like structures were visualized in the other regions of interest or in all control fetuses.

### Histochemical analyses of olfactory pathways

#### Monoaminergic content in the olfactory bulb

In order to highlight potential neurochemical alterations, the tissue contents of the biogenic monoamines serotonin (5-HT), dopamine (DA) and noradrenaline (NA) in the OB of control and exposed fetuses were analyzed. Concerning the serotoninergic system, neither tryptophan (TRP) nor Kynurenine (K) levels were significantly different from controls (see Table 1). 5-HT levels were decreased in exposed fetuses (*p* = 0.023); the 5-HT/TRP ratio of exposed fetuses was slightly lower than in controls, although not significant (*p* = 0.09). Concerning the 5-Hydroxyindoleacetic acid (5-HIAA) levels and 5-HIAA/5-HT ratio, no differences were observed between the two groups (Table 1).

**Table 1:** Effects of DE exposure on the serotonergic pathway of GD28 fetuses

	Control		Exposed		Effects of DE exposure		Effects of Sex		Exposure × Sex	
	Male (*n =* 4)	Female (*n =* 4)	Male (*n =* 4)	Female (*n =* 4)	F (1,15)	*p*-value	F (1,15)	*p*-value	F (1,15)	*p*-value
Trp (fmol/mg)	77,941 ± 4357.6	77,737 ± 9898.7	66,086 ± 7164.3	79,000 ± 7635.3	0.139	0.716	0.983	0.343	0.582	0.462
K (fmol/mg)	14,844 ± 689.1	13,875 ± 1144.6	12,028 ± 1334.5	12,611 ± 1312.6	0.669	0.431	0.000	0.995	0.049	0.829
5-HT (fmol/mg)	1718 ± 228.3	1546 ± 151.4	1081 ± 158.8	1187 ± 133.4	6.972	0.023*	0.046	0.833	0.724	0.413
5-HT/Trp	0.022 ± 0.004	0.020 ± 0.001	0.017 ± 0.003	0.015 ± 0.002	3.457	0.090	0.628	0.445	0.081	0.782
5-HIAA (fmol/mg)	170.0 ± 39.16	163.8 ± 32.29	197.9 ± 73.28	156.4 ± 18.62	0.244	0.631	0.199	0.664	0.136	0.720
5-HIAA/5-HT	0.098 ± 0.016	0.117 ± 0.011	0.184 ± 0.056	0.136 ± 0.023	0.822	0.384	0.455	0.514	0.638	0.441

Basal rates of Tryptophan (Trp), Kynurenine (K), Serotonin (5-HT) and 5-Hydroxyindoleacetic acid (5-HIAA), along with the correspondent ratios. Results were expressed as fentomoles/milligram of fresh tissues (fmol/mg). Results are shown as mean ± SEM. *p < 0.05 represented a significant difference between each group. 5-HT levels were decreased in exposed fetuses compared to controls. No significant difference existed between groups regarding the other variables of interest

For catecholamine levels, neither NA nor its main metabolite 3-Methoxy-4-hydroxyphenylglycol (MHPG) were significantly changed when compared to controls. However, the MHPG/NA ratio in exposed fetuses was higher when compared to controls (*p* = 0.027) (Table 2). Interestingly, the latter ratio revealed a sex-dependent difference between the two groups (*p* = 0.04) with no interaction between sex and DEP exposure (*p* = 0.289).

**Table 2:** Effects of DE exposure on the catecholamine system of GD28 fetuses

	Control		Exposed		Effects of DE exposure		Effects of Sex		Exposure × Sex	
	Male (*n =* 4)	Female (*n =* 4)	Male (*n =* 4)	Female (*n =* 4)	F (1,15)	*p*-value	F (1,15)	*p*-value	F (1,15)	*p*-value
DA (fmol/mg)	274.9 ± 27.59	276.1 ± 37.85	389.7 ± 45.00	341.9 ± 41.79	3.502	0.088	0.525	0.484	0.262	0.619
DOPAC (fmol/mg)	212.1 ± 56.13	212.1 ± 43.34	346.0 ± 122.38	460.3 ± 84.89	2.451	0.146	0.453	0.515	1.308	0.277
HVA (fmol/mg)	222.6 ± 32.11	280.4 ± 32.21	514.1 ± 71.17	412.0 ± 134.76	2.761	0.125	0.357	0.562	0.311	0.588
DOPAC/DA	0.789 ± 0.240	0.789 ± 0.147	0.857 ± 0.247	1.33 ± 0.104	0.738	0.409	1.644	0.226	0.612	0.115
HVA/DA	0.810 ± 0.084	1.066 ± 0.214	1.315 ± 0.068	1.158 ± 0.247	0.411	0.534	0.016	0.901	0.612	0.451
(DOPAC+HVA)/DA	1.599 ± 0.290	1.855 ± 0.303	2.173 ± 0.307	2.488 ± 0.348	0.854	0.375	0.879	0.369	0.557	0.471
NA (fmol/mg)	414.7 ± 14.90	488.8 ± 45.88	304.0 ± 54.32	437.1 ± 145.76	1.945	0.191	1.808	0.206	0.431	0.525
MHPG (fmol/mg)	126.9 ± 17.15	94.45 ± 24.17	135.0 ± 31.76	137.9 ± 78.42	0.613	0.450	0.099	0.759	0.071	0.794
MHPG/NA	0.304 ± 0.36	0.192 ± 0.039	0.469 ± 0.102	0.277 ± 0.091	6.466	0.027*	5.416	0.040*	1.241	0.289

Basal rates of Dopamine (DA), 3,4-Dihydroxyphenylacetic acid (DOPAC), Homovanillic acid (HVA), Noradrenalin (NA), and 3-Methoxy-4-hydroxyphenylglycol (MHPG), along with the correspondent ratios. Results were expressed as fentomoles/milligram of fresh tissues (fmol/mg). Results are shown as mean ± SEM. *p < 0.05 represented a significant difference between each group. Neither NA, nor its main metabolite MHPG, was significantly changed when compared to controls. However, the MHPG/NA ratio in exposed fetuses was higher when compared to controls. DA levels were slightly higher in exposed fetuses, even if not significant. No significant difference existed between groups regarding the other variables of interest

Concerning the dopaminergic pathway, DA levels tended to be higher in exposed fetuses (*p* = 0.088). Neither the levels of its metabolites (3,4-Dihydroxyphenylacetic acid, DOPAC and Homovanillic acid, HVA), nor the ratios (HVA/DA and DOPAC/DA) or DA turnover were significantly affected by the gestational exposure (Table 2).

#### Bulbar intrinsic DA-cell subpopulations

In order to establish a link between the anatomical and the neurochemical disturbances of the olfactory system observed at GD28, the intrinsic DA-cells subpopulation within the glomerular cell layer was analyzed through the quantification of the Tyrosine Hydroxylase (TH)-specific labeled cells.

There was no significant differences in the number of TH^+^ cells in the fetal OBs between the two groups [means = (7.12 ± 0.97)× 10^− 5^ vs. (7.48 ± 1.40)× 10^−5^cells / surface unit (a.u) in controls and exposed, respectively; *p* = 0.79)] (Table 3). This result was confirmed with another quantitative estimation using immunofluorescence analysis; the latter study showed no difference in the number of TH^+^-cells, DAPI^+^-cells or TH^+^-cell /DAPI^+^-cell ratio between the two groups (data not shown).

**Table 3:** Quantification of TH-labeled cell number in GD28 olfactory bulbs

	Control		Exposed		Effects of DE exposure		Effects of Sex		Exposure × Sex	
	Male (*n =* 4)	Female (*n =* 4)	Male (*n =* 8)	Female (*n =* 5)	F (1,20)	*p*-value	F (1,20)	*p*-value	F (1,20)	*p*-value
TH-labeled cell (10 × −5cells / surface unit, a.u)	7.99 ± 1.49	6.25 ± 1.48	6.57 ± 1.91	8.94 ± 2.41	0.074	0.790	0.020	0.888	1.013	0.329

Quantification of the number of TH+ labeled cells in control and exposed samples. *p < 0.05. No significant difference existed between groups

#### TH immunochemistry and acetylcholinesterase activity

**Fig. 4:**
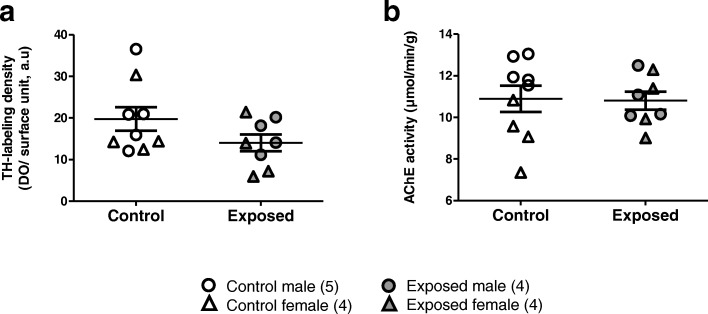
Quantification of dopaminergic and cholinergic inputs into GD28 olfactory tubercle. **a** Relative mean density of TH+ labeled fibers in the olfactory tubercle. The quantitative estimation did not show any significant difference between the two groups. **b** Relative means density of AChE activity in the olfactory tubercle. There was no significant difference between the two groups regarding the DE exposure. However, we observed a sex-dependent difference between the two groups, which interacted significantly with DE exposure

Intensity of TH-staining, along with measurements of Acetylcholinesterase (AChE) activity were measured in glomerular cells of the OB in order to analyze the effect of DE exposure on the neuromodulatory inputs to the OB (Fig. 4). TH analysis did not show any significant difference between the two groups (*p* = 0.143) (Fig. 4a). The statistical analysis of AChE activity (Fig. 4b) did not reveal any significant effect of DEP exposure (*p* = 0.942). However, we observed a sex-dependent difference between the two groups (*p* = 0.013), and an interaction between fetus sex and maternal DEP exposure (*p* = 0.033) (Table 4). This result, thus, highlights a sex-dependent vulnerability upon DEP exposure, with males presenting a decrease and females presenting an increase of AChE activity.

**Table 4:** Quantification of dopaminergic and cholinergic inputs in GD28 olfactory tubercles

	Control		Exposed		Effects of DE exposure		Effects of Sex		Exposure × Sex	
	Male (*n =* 4)	Female (*n =* 4)	Male (*n =* 8)	Female (*n =* 5)	F (1,16)	*p*-value	F (1,16)	*p*-value	F (1,16)	*p*-value
TH-labeling density (DO/surface unit, a.u)	21.27 ± 4.65	17.86 ± 4.84	15.89 ± 2.34	12.14 ± 4.11	2.461	0.143	1.080	0.319	0.001	0.980
AChe activity (μmol/min/g)	12.25 ± 0.34	9.21 ± 0.83	10.96 ± 0.85	10.66 ± 0.85	0.006	0.942	8.462	0.013*	5.773	0.033*

Relative mean density of TH+ labeled fibers in the olfactory tubercle. The quantitative estimation did not show any significant difference between the two groups

Relative mean density of AChE activity in the olfactory tubercle. There is no significant difference between the two groups regarding the DE exposure. However, we observed a sex-dependent difference between the two groups, which interacted significantly with DEP exposure. *p < 0.05

### Olfactory-guided behavior is disturbed by DEP exposure at PND2

In order to highlight a possible connection between the anatomical and the neurochemical disturbances observed in GD28 and putative early olfactory behavioral alterations, a 2MB2 odor-guided behavior test was run in PND2 pups born to exposed or non-exposed dams.

**Fig. 5:**
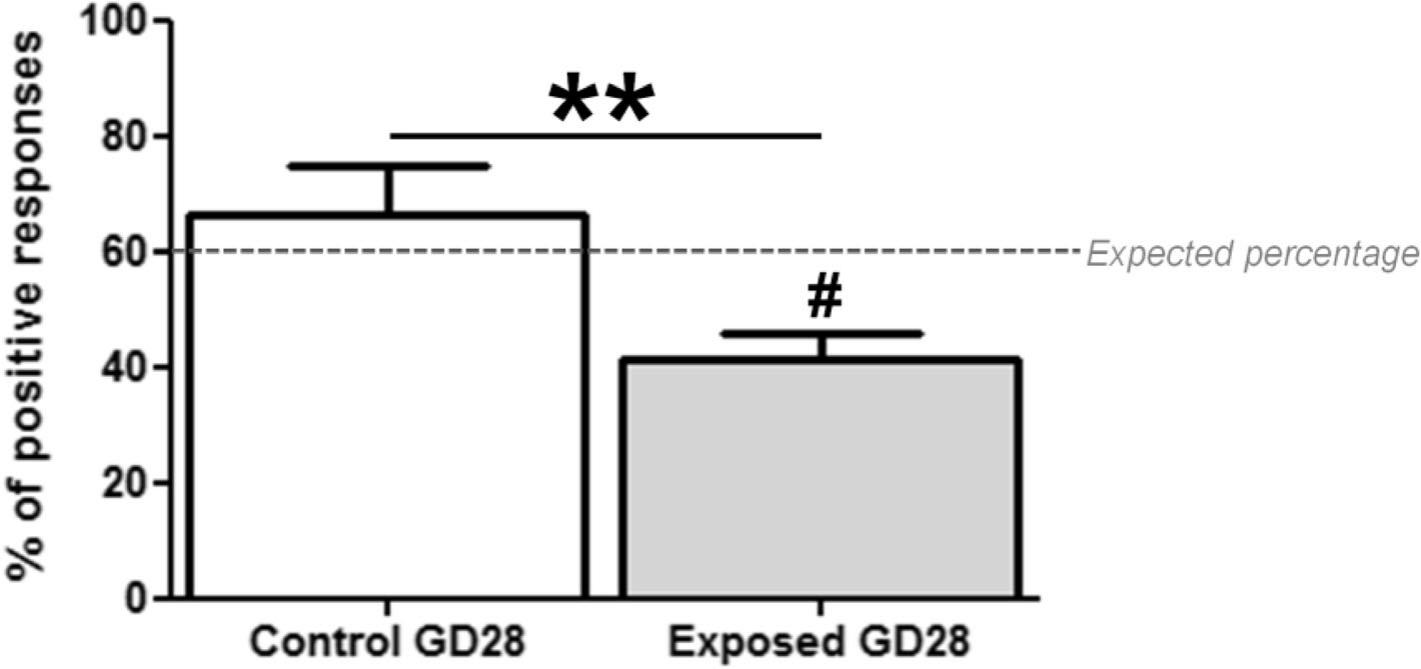
Percentages of positive behavioral responses following to the presentation of the rabbit mammary pheromone 2MB2. Results are expressed as mean ± SEM for each group. # *p* < 0.01 represented a significant difference between the groups and the expected percentage (60%). The percentage of positive responses in exposed fetuses (41%) was significantly lower, whereas that of control fetuses (66%) was not significantly different. **p < 0.01 represented a significant difference between the two groups. The comparison of the percentages showed a significant difference between the two groups

Pearson’s Chi-square analysis indicated that the observed percentage of positive responses to 2MB2 in control pups (66%) was not significantly different from previous reports [67] (Pearson’s Chi-square, df = 0.5269; *p* = 0.4679) (Fig. 5, 60% of expected positive response, dotted line), but it was significantly lower in exposed pups (41%) (Pearson’s Chi-square, df = 9.1667; *p* = 0.0025). Interestingly, the percentage of positive responses to 2MB2 in exposed pups was significantly lower than in control pups (Pearson’s Chi-square, df = 7.032; *p* = 0.008) (Fig. 5).

## Discussion

Using the animal model of controlled nose-only inhalation developed to analyze feto-placental development [62], we have shown that maternal exposure to DE throughout gestation leads to an accumulation of particle-like structures in fetal olfactory tissues, which appears to be associated with alterations in the neuroanatomical organization of this system. Moreover, such DE exposure affects monoaminergic neurotransmission in the OB and the olfactory-based behaviors connected with nutrient intake at birth. To our knowledge, this is the first study assessing the neuroanatomical nose-brain continuum around birth, even though a direct link between the anatomical and neurochemical disturbances of the olfactory system observed at the fetal stage and the behavioral alterations registered in postnatal early life remains to be demonstrated. These data confirm and expand previous results suggesting that UFPM are of great concern given their particular characteristics (i.e. size, surface charge, surface coating, chemical composition,..), which have been argued to determine their capacity to access organs and exert tissue alterations [68–73]. They thus are suspected to represent a class of particles with serious health effects compared to PM10 and PM2.5 [74, 75], and for which strong epidemiological evidence is not available yet due to the lack of exposure data [76]. Although we used a polluted air enriched in nanosized particles, we cannot exclude that the observed effects are also linked to the presence of other chemical components, known to be associated to neurodevelopmental deficits in several cases of gestational exposition [77, 78]. The inhalation model of exposure was monitored for volatile compounds including carbon monoxide and nitric oxide products with levels of 7 and 36 μg/m^3^, respectively. Such values were largely under the levels used for traffic regulation in urban areas, suggesting the observed effects on olfactory system to be more related to NP exposure than to gaseous components. DE is composed of hundred chemical substances including among them Polycyclic Aromatic Hydrocarbons which have been demonstrated to be neurotoxic [79]. Whereas PAHs may contribute for a part to the effects observed here, it was not possible in the present study to measure their levels of concentration in the brain and the olfactory bulb.

### Gestational inhaled diesel engine exhaust can reach fetal olfactory structures

We have demonstrated the presence of NP-like structures in various regions of the fetal olfactory system, from the periphery to the central OB structures, whose diameters were estimated between 5 and 20 nm depending on the structure (mean diameter of inhaled particles during exposure was 69 nm). To our knowledge, the current results demonstrate for the first time a possible nasal route of fetal exposure after maternal nose-only inhalation of DE in controlled conditions mimicking the fetal exposure encountered by humans in everyday life. So far, most studies describing particles in olfactory tissues have been performed in conditions of sustained exposure to non-controlled air pollutants, either in a chronic or acute highly concentrated environment in rodents [80, 81], dogs [13] or human [15]. However, it was not formally confirmed that these particles originated from the diesel exposure, since further analyses to determine the chemical composition of these NP-like structures are hindered by the important carbon content of DE. Moreover, so far, it has not been possible to study the chemical composition of their corona, although it could provide insight into mechanisms by which these particles access biological tissues [82, 83].

Even if it was not demonstrated how the inhaled particles can translocate to the fetal olfactory system, it is quite likely that the NP could have been transported to the fetal olfactory organs through the systemic circulation since Valentino and colleagues have shown, on the same animals, that NPs were able to cross the maternal lung barrier and reach fetal erythrocytes across the placenta [62, 64]. Another possibility to explain the presence of NP-like particles at the level of the olfactory cilia bathing the nasal mucus is a direct translocation of NP from the amniotic fluid. It would be interesting to confirm the presence of NP in the amniotic liquid of exposed dams, as observed for tobacco substances in pregnant smoking women [84], in order to have an insight on the mechanisms by which fetuses are exposed to the particles, all mechanisms not being exclusive.

Interestingly, even if we observed free NP-like structures in the olfactory cilia, most were concentrated in lysosome-like vesicles throughout the olfactory structures. Such vesicles were not observed in the placenta of exposed rabbits, suggesting that the process might be specific to olfactory structures [62]. Whether they are associated with cell-specific uptake mechanisms such as lysosomal deposition or caveolae remains to be characterized. As UFPM have already been observed in caveolae of endothelial cells and in luminal red blood cells in myocardial capillaries of 1-year-old dogs from highly polluted cities [85], our observations also fit with an ongoing hypothesis of transportation of inhaled NPs through a process called “transcytosis” [82].

### A controlled repeated gestational exposure to DE alters the bulbar neuromodulation in exposed fetuses

We observed disturbances in the nerve fascicles arising from the olfactory epithelium, along with a decrease in axonal density and in cellular components around glomeruli in exposed fetuses. Such structural alterations are unlikely to be the consequence of a peripheral perturbation in the number and/or guidance of OSN projections to the OB, since no modification in the density of olfactory cells was observed in the OM. Interestingly, the observed heterogeneous hypertrophy of OSN axons was reminiscent of a “swelling” phenomenon, suggesting the onset of inflammatory processes of the cells, that has been shown in other neuronal cells following gestational DE exposure [37, 39]. Furthermore, we noticed evidence of ultrastructural modifications of nuclei in various olfactory cell types, suggesting early signs of cell death in OM and OB. Altogether, these observations suggest that DE impact the cellular homeostasis in olfactory structures at a timing encompassing an important window of developmental neurogenesis and could, thus, interfere with fetal neurobiological development.

Associated with these ultrastructural alterations, we observed bulbar neuromodulatory disturbances, with a decrease in 5-HT levels and an increase in NA turnover in the OB of exposed fetuses. Moreover, a sex-dependent alteration in cholinergic inputs to the OB was shown. Furthermore, although neither bulbar intrinsic DA-cell subpopulations nor dopaminergic projection inputs to the OB seemed to be affected by DE exposure, a tendency of increase in bulbar DA levels was observed.

Our data thus suggest that alterations of the centrifugal olfactory input precede a bulbar dopamine disturbance, which is not inconsistent with an ongoing hypothesis suggesting that the bulbar dopaminergic alterations are a compensatory plastic response following reduced neuromodulatory innervations [60].

Given that the noradrenergic, serotoninergic, dopaminergic and cholinergic projections originate from central structures, the neurochemical alterations might not only concern olfactory processes, but also more integrative ones. Such early perturbations on fetal neural network might also persist later in life and lead to neurocognitive disorders related to monoaminergic systems [41–45, 86]. Further analyses need to be performed in order to see if and how DE exposure alters the modulatory pathways in other brain regions and, therefore, to further substantiate the toxic effects of DE inhalation on the CNS.

Interestingly, sex-dependent neurochemical disturbances in the OB and Tu of exposed fetuses were observed. Such a possible link between gestational air pollution exposure and sex-dependent alterations of the CNS has already been pointed out in recent studies, with males being often more affected by environmental insults than females [34, 47, 48, 87]. However, to our knowledge, this is one of the rare studies comparing monoamine fetal disturbances between sexes. Indeed, most studies have been performed on in utero exposed male adult mice only to show imbalances in the monoaminergic systems related to various neurocognitive disorders [41–44]. Moreover, behavioral evaluations on both male and female mice have shown that prenatal exposure to UFPM induces more severe consequences on males [88]. Furthermore, male offspring prenatally exposed to both airborne pollution and maternal stress during late gestation were cognitively impaired, suggesting that males could be more sensitive to the adverse CNS changes after DE exposure [34]. Nevertheless, the PM-induced sex-dependent CNS changes are quite complex [88] and require further investigation.

Overall, our data suggest that early life exposure to airborne pollution may affect the fetal brain development and might predispose the progeny to neurodevelopmental diseases later in life, which is in agreement with the recent work of Calderon-Garcidueñas and colleagues in children and young adults [89].

### A controlled repeated gestational exposure to DE leads to disturbance in odor sensitivity in rabbit offspring

The behavioral performance of rabbit neonates clearly revealed early olfactory behavioral alterations at birth following a repeated gestational exposure to DE. Rabbit newborns, for that matter, constitute an interesting model system given that they display innate behaviors mainly governed by olfactory cues to feed at this stage, through the stereotyped behavioral pattern in response to the rabbit mammary pheromone 2-Methyl-3-butyn-2-ol [90, 91] through the main olfactory system. In utero DE exposed pups exhibited a decreased response to 2MB2, tested at a single commonly used concentration [90]. Since the newborn response to the mammary pheromone follows a concentration-dependent bell-shaped curve, with an optimal responsiveness between 10^− 8^ and 10^− 6^ g/ml [67], the low response at 5.10^− 8^ mg/ml could be due to a shift of the bell-shaped curve response to lower or higher concentrations, thus indicating either a worsened or a better olfactory threshold in exposed animals. However, the morphological and neurochemical alterations observed at GD28 in the main olfactory system favors an impairment of the neurobehavioral development of newborns, even though a direct link between the anatomical and functional disturbances of the olfactory system observed in GD28 and the behavioral alterations registered at PND2 remains to be demonstrated [90, 92]. Moreover, it is possible that the structural and neurochemical alterations not only concern olfactory processes, but also more integrative ones, since gestational exposure to DE was demonstrated to induce various neurocognitive disorders related to monoaminergic systems [41–45, 86]. In fact, because of the known anatomical and functional continuum between the olfactory system and the rest of the brain, there could be olfactory transport of toxic materials, including particulate matter, into various regions of the brain connected with several aspects of other motivated behaviors [15, 93, 94]. Besides, no difference in the growth of pups was observed until weaning (Charlier M. and Rousseau D., unpublished data) suggesting that exposed pups have an efficient suckling behavior, despite the modification in 2MB2 pheromone response (not shown).

## Conclusion

The present study describes the neurodevelopmental toxic effects on the olfactory system of a controlled daily gestational exposure to DE on a human hemodichorial placentation animal model in conditions mimicking urban human exposure. Overall, our findings strongly suggest that prenatal exposure to DE in rabbit fetuses allows translocation of nanoparticles towards fetal nervous system structures with potential pathological effects on the nervous system. Such exposure affects the neuro-olfactory development of rabbit offspring, and alters early olfactory-based behaviors. Because of the known anatomical and functional continuum between the olfactory system and the rest of the brain, such early alterations could be indicative of disturbances in higher integrative structures. These data confirmed that prenatal phase should be considered as an important window for brain development, during which there is an elevated susceptibility to environmental insults [24, 78, 95]. Preventing exposure of pregnant women to a polluted environment should, therefore, be taken into account by public authorities.

## Methods

### Animal exposure

**Fig. 6:**
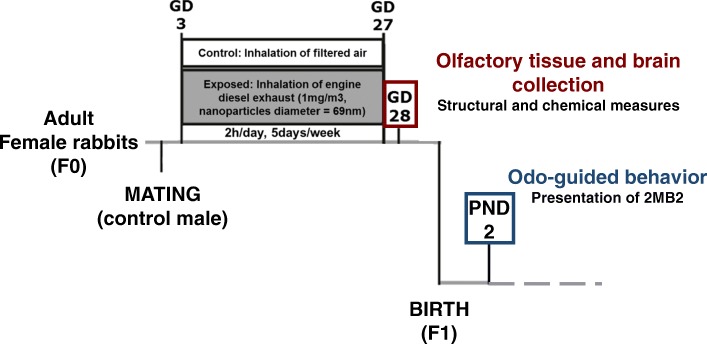
Experimental protocol. Pregnant New-Zealand white female rabbits were exposed by nose-only inhalation to either clean air (control group; *N* = 14) or DE containing diluted DEP (1 mg/m^3^) (exposed group; *N* = 14) for 2 h/day, 5 days/week, from gestational day 3 (GD3) to day 27. At GD28, 12 dams (*N* = 5 controls; *N* = 7 exposed) were euthanized and their fetuses sacrificed by decapitation. Fetal olfactory mucosa (OM), hemi-olfactory bulbs (OB) and whole brains were dissected. Random samples of the latter structures were chosen for structural and chemical measures Eighteen exposed or control dams (*N* = 9 each) gave birth to F1 offspring. The day of birth was settled as postnatal day 0 (PND0). At PND2, offspring were examined for their odor-guided behavior in response to the presentation of the rabbit mammary pheromone 2-Methyl-3-butyn-2-ol (2MB2). They were then raised in control conditions in order to study long-term and intergenerational effects of this gestational DE exposure.

The present study is part of a larger program funded by the ANR (ANR-13-CESA-0011-EPAPP). The experimental procedure for animal exposure has been previously detailed and approved by the French ethical committee N°45 under the number N°12/102 [62]. Briefly, twenty-eight pregnant New-Zealand white female rabbits (INRA1077 line, 1-year old) (F0) were exposed by nose-only inhalation to either clean air (control group; *N* = 14) or diluted DE (1 mg/m^3^) (exposed group; *N* = 14) for 2 h/day, 5 days/week, from gestational day 3 (GD3) to day 27 (i.e., 20 days over a 31-day gestation) (Fig. 6, adapted from [62]).

DE exposure was performed with the Mobile Ambient Particle Concentrator Exposure Laboratory [96], connected to a 25KVA Loxam engine, with a 500 nm particle filter. The measured components of the DE exposure mixture used in the present experiment were analyzed elsewhere and their NP content displayed an average size of 69 nm [62].

### Experimental procedure

At GD28, 12 dams (*N* = 5 controls; *N* = 7 exposed) were euthanized and their fetuses counted, weighed individually and euthanized by decapitation. Skilled staff using well-maintained equipment ensured the rapid death of each animal and carried out the manipulation under appropriate conditions. All experiments were performed by confirmed experimenters or with their help. F1 fetuses were sexed by visual observation of their internal genital organs (confirmed by a second sexing by qPCR approach). Fetal olfactory mucosa (OM), hemi-olfactory bulbs (OB) and whole brains were dissected and nervous tissues were weighed. We observed no difference in total brain or OB weight between exposed and not exposed fetuses (not shown). Random samples of the latter structures were snap frozen in liquid nitrogen or isopentane for subsequent anatomical and chemical measures. Non-frozen OB and OM were randomly assigned to histological, immunological or transmission electron microscopy (TEM) experiments.

Eighteen exposed or control dams (*N* = 9 in each group) gave birth to F1 offspring. The day of birth was settled as postnatal day 0 (PND0). At PND2, offspring were examined for their odor-guided behavior in response to the presentation of the rabbit mammary pheromone 2-Methyl-3-butyn-2-ol (2MB2) as published elsewhere [90]. They were then raised in control conditions in order to study long-term and intergenerational effects of this gestational DE exposure (as described previously [62]).

### Olfactory system measurements

#### TEM analysis of the olfactory system

Fetal OM and OB samples collected randomly from 2 exposed and 2 control dams (one male and one female per litter; *N* = 4 in each experimental group) were fixed in 2% glutaraldehyde overnight at 4 °C. Samples were contrasted with 0.5% OTE (Oolong Tea extract) in cacodylate buffer (20 min, 37 °C), fixed in 1% OsO4 containing 1.5% ferrocyanide (20 min, 37 °C), dehydrated using a series of ethanol dilutions (5 min each, 37 °C) and acetone (10 min, 37 °C) and embedded in Epon resin using a microwave (60 min, 50 °C). Sections (1 μm) were stained with methylene Blue/Azure II and examined with an Olympus microscope. Ultrathin sections (75 nm) were stained with lead citrate and examined with HITACHI HT7700 transmission electron microscope at 80KeV on MIMA2 platform (https://www6.jouy.inra.fr/mima2). For each sample, several blocks were cut (*N* = 2) and at least 4 grids/block were observed by a blind experimenter. Images were selected to show a representative view of the alterations in the olfactory tissues.

#### Bulbar HPLC dosage

The levels of DA, 5-HT, NA, and their respective metabolites were quantified using high performance liquid chromatography (HPLC) on crushed OB samples. Prior to analysis, randomly chosen right OBs from control or exposed fetuses (*N* = 4 male and *N* = 4 female per group) were weighted, crushed in 400 μL of 0.2 M perchloric acid and centrifugated at 22,000 g for 20 min at 4 °C. The supernatants were collected and filtered through a 10 kDa membrane (Nanosep, Pall) by centrifugation at 7000 g (30 min). Then, a 20 μL aliquot of each sample was analyzed for 5-HT and 5-HIAA by fluorometric detection (Kema). The amounts of catecholamines (DA and NA) and of their main metabolites (DOPAC and HVA for DA; MHPG for noradrenaline) were measured by electrochemical detection on a serial array of coulometric flow-through graphite electrodes (CoulArray, ESA). Analysis, data reduction, and peak identification were fully automated. Results were expressed as femtomoles/milligram of fresh tissues [97, 98]. Results are shown as mean ± SEM.

#### Bulbar tyrosine hydroxylase immunohistochemistry and image analysis

Randomly chosen fetal OBs (*N* = 4 male and *N* = 4 female control fetuses; *N* = 8 male and *N* = 5 female exposed fetuses) were fixed during 24 h in 4% paraformaldehyde (PFA) at 4 °C and cryoprotected with a 30% sucrose solution in PBS for 3 days at 4 °C. Tissues were thereafter embedded in tissue-TEK and store at − 80 °C.

Serial sections (16 μm) of GD28 OBs were mounted on Super Frost slides (Roth-Sochiel, Lauterbourg, France) and kept at − 80 °C until use. The slices were dried at room temperature for 20 min. The endogenous peroxidase activity was inhibited by H_2_O_2_ (0.3%) for 30 min. Sections were then blocked with 1% BSA and normal horse serum for 30 min and incubated overnight at 4 °C in presence of mouse monoclonal primary anti-tyrosine hydroxylase antibody (TH, catalogue #T1299; Sigma, St. Louis, MO, 1:5000 in 0.3% Triton X-100). Samples were then incubated for 30 min at room temperature with biotinylated anti-mouse IgG (Vectastain Elite ABC Kit, PK-6102, Vector laboratories, Burlingame, USA), followed by avidin-biotin-peroxidase complex (Vectastain Elite ABC Kit, PK-6102, Vector laboratories, Burlingame, USA) that was used according to the supplier’s instructions. The slices were revealed for 10 min using 3′,3-diaminobenzidine (DAB) substrate kit for visualization (SK-4100, Vector laboratories, Burlingame, USA), resulting in a gray-black reaction product. The reaction was stopped with deionised water. The sections were then dried and coverslipped with Eukitt mounting medium (catalogue #03989; Sigma, St. Louis, MO) and kept in the dark until image capture.

Stained sections were examined using a light upright optical microscope (Nikon Eclipse Ni-U) at 4x and 40x magnifications (Nikon France, Champigny-sur-Marne, France) and quantified using the NIS-Elements Br software (Nikon). A blind experimenter quantified all images.

Quantification of the number of TH-immunoreactive cells (TH^+^) was carried out by examination of 2 non-adjacent sections per fetus photographed with the 4x objective, on 4 independent immunohistochemistry experiments. After application of an optimal threshold ([30, 170]) to exclude the non-specific labeling and the background, the quantification of TH-immuno-labeled cells was fully automated. The number of TH^+^ cells was then normalized to the selected area (ROI) size. Two independent ROIs per section were analyzed (i.e., four measures per animal).

### Brain histological and histochemical measurements

#### Tissue preparation

Randomly chosen fetal brains (*N* = 5 male and *N* = 4 female control fetuses; *N* = 4 male and *N* = 4 female exposed fetuses) were rapidly frozen in cooled isopentane at − 34 °C and subsequently stored at − 80 °C. Twenty μm-thick coronal sections were serially cut on a cryostat, collected on gelatin-chrome alum-coated slides and stored at − 80 °C until histological and histochemical processing.

#### Brain tyrosine hydroxylase and tryptophan hydroxylase immunohistochemistry and image analysis

Slices were dried at room temperature for 20 min. Sections were incubated in 4% formaldehyde for 30 min. The immunohistochemical protocol has been described above (5.3.3). Sections were incubated overnight at 4 °C in presence of mouse monoclonal primary anti-tyrosine hydroxylase antibody (TH, catalogue #T1299; Sigma, St. Louis, MO, 1:5000 in 0.3% Triton X-100) or in presence of mouse monoclonal primary anti-tryptophan hydroxylase antibody (TrpH, catalogue #T0678; Sigma, St. Louis, MO, 1:2000 in 0.3% Triton X-100). The slices were revealed for 3 min using DAB substrate kit.

Analysis of TH and TrpH stained sections was carried out with a BIOCOM computer-assisted image analysis system (Les Ulis, France), in which optical density readings were performed. In absence of an available rabbit brain atlas, anatomical structures were defined according to the Paxinos and Watson stereotaxic atlas of the rat brain (6th edition, 2007). The total labeling was measured by taking the mean of two optical densities on the same slice and on two successive slices. The mean non-specific labeling (measured on a region without TH or TrpH labeling) was subtracted of the total labeling. The measures were carried out on the olfactory tubercle (Tu), a structure by which central neuromodulatory inputs enter the OB.

#### Histochemical measurement of acetylcholinesterase activity

Slides were incubated for 12 h in 180 mL of stock solution (see below) to which had been added 208 mg of S-acetylthiocholine iodide and 5.4 mg ethopropazine. The slides were rinsed with deionized water and developed for 10 min in 1% sodium sulfide in phosphate buffer 0.1 M at pH 7.5. They were then rinsed three times for 5 min each with deionized water and immersed in 4% paraformaldehyde in phosphate buffer 0.1 M for 30 min, and then rinsed with distilled water (3 times; 5 min each). Subsequently, slides were dehydrated in series of ethanol baths (50, 70, 96, 100%) for 3 min, and cleared with toluene twice for 5 min. Slides were then coverslipped with Eukitt mounting medium (catalogue #03989; Sigma, St. Louis, MO). The stock solution was a 50 mM sodium acetate buffer at pH 5.0 which was made 4.0 mM with respect to copper sulphate and 16 mM with respect to glycine. This was done by adding 6.8 g of sodium acetate, 1.0 g of copper sulphate crystals, and 1.2 g of glycine to 1.0 L of water and lowering the pH to 5.0 with HCl.

Standards for specific acetylcholinesterase activity were prepared from whole brain homogenates of adult rabbits. Enzyme activity of brain homogenates was biochemically measured by spectrophotometry according to the protocol described by Ellman et al. and modified by Dumont et al. [99, 100]. Briefly, brain homogenate (pH = 8) was diluted with 5–5′-dithiobis-2-nitrobenzoate acid (DTNB, 0.01 M) and acetylthiocholine iodide (75 mM). The thio-choline, formed during hydrolysis of acetylthiocholine, rapidly reacts with DTNB to release a colored 5-thio-2-nitrobenzoate anion with maximum absorption at 412 nm. The specific enzymatic activity of acetylcholinesterase was calculated from the linear slope of the curve obtained and was 8.41 μmol/min/g of tissue. Brain homogenate sections of variable thickness were used to cover the entire range of activity measured in the different brain structures. Under our experimental conditions, the staining intensity was highly correlated to the thickness of the standard sections (linear function with *r* = 0.997). Analysis of acetylcholinesterase stained sections was carried out with a BIOCOM computer-assisted image analysis system (Les Ulis, France), in which optical density readings were converted by means of standards into enzymatic activity values expressed in μmol/min/g of tissue. Anatomical structures were defined according to the Paxinos and Watson stereotaxic atlas of rat brain (6th edition, 2007). At least 3 optical density readings were taken of labeled regions to obtain a homogeneous evaluation.

### Olfactory-guided behavior

The olfactory sensitivity of exposed or control pups was tested at PND2. 117 pups (*N* = 62 control; *N* = 55 exposed) born to 15 dams (*N* = 8 controls, *N* = 7 exposed) were examined for their odor-guided behavior in response to the presentation of 2MB2 (catalogue #136816; Sigma-Aldrich, Saint-Quentin Fallavier, France) as previously described [90, 91]. To standardize the feeding status of newborn animals, the access of the mother to the nest was blocked at 5 pm the day before the experiment and pups were individually tested at 8 am. The test consisted on taking each pup in a gloved hand with nest material, with its head left free to move. When the pup reached a state of relative immobility, the extremity of a glass-stick (length×diameter: 15 × 0.3 cm) carrying the 2MB2 stimulus (final concentration of 5.10^− 8^ g/ml in water, freshly prepared each day) was positioned 0.5 cm in front of its muzzle without contact with its skin or hair. The stimulus was presented for 10s and individual scores (yes or no) were recorded. Pups were considered to positively respond when they displayed searching movements, consisting of vigorous horizontal and vertical scanning of the head, after maximal stretching of the neck toward the stimulus. Results were expressed as the percentage of pups displaying searching and oral grasping responses when exposed to the molecule (i.e., positive responses). The final concentration used here has been reported to induce 60% of positive responses in control animals [90].

Thereafter, pups were immediately put back in the nest. The whole litter was tested in less than 15 min and the mother was allowed access to the nest at the end of the experiment. The glass-sticks were rinsed twice with pure ethanol and distilled water, and then dried between each pup.

### Statistical analysis

For each variable measured in the present study, a Levene test for homogeneity of variances was performed. Thus, given that homogeneity of variance was assumed for most of the variables, a general linear model (GLM) was used for statistical analysis, adjusting the litter as a confounding variable. A Pearson chi-square procedure was used to analyze the percentage of animals that responded to the mammary pheromone 2MB2, without considering the litter or the sex, which was not checked at this step. Significance was set at *p* < 0.05, tendency at *p* < 0.1. All statistical analyses were carried out using SPSS 16.0 software (SPSS Inc., Chicago, IL, USA).

## Abbreviations

2MB2: 2-Methyl-3-butyn-2-ol
5-HIAA: 5-Hydroxyindoleacetic acid
5-HT: Serotonin
AChE: Acetylcholinesterase
AD: Alzheimer’s disease
CNS: Central nervous system
DA: Dopamine
DE: Diesel exhaust
DEP: Diesel exhaust particles
DOPAC: 3,4-Dihydroxyphenylacetic acid
F1: First generation
GD: Gestational day
GLM: General linear model
Gr: Granule cells
HVA: Homovanillic acid
K: Kynurenine
MHPG: 3-Methoxy-4-hydroxyphenylglycol
NA: Noradrenaline
NP: Nanoparticles
OB: Olfactory bulb
OEC: Olfactory ensheating cells
OM: Olfactory mucosa
OSN: Olfactory sensory neurons
PD: Parkinson’s disease
PG: Periglomerular cells
PM: Particulate matter
PND: Postnatal day
ROI: Selected area
TEM: Transmission Electron Microscopy
TH,: Tyrosine Hydroxylase
TRP: Tryptophan
TrpH: Tryptophan Hydroxylase;
UFPM: Ultrafine particle matter
